# Putting ChatGPT vision (GPT-4V) to the test: risk perception in traffic images

**DOI:** 10.1098/rsos.231676

**Published:** 2024-05-29

**Authors:** Tom Driessen, Dimitra Dodou, Pavlo Bazilinskyy, Joost de Winter

**Affiliations:** ^1^ Delft University of Technology, Delft, Zuid-Holland, The Netherlands; ^2^ Eindhoven University of Technology, Eindhoven, Noord-Brabant, The Netherlands

**Keywords:** risk perception, traffic, image-to-text, ChatGPT

## Abstract

Vision-language models are of interest in various domains, including automated driving, where computer vision techniques can accurately detect road users, but where the vehicle sometimes fails to understand context. This study examined the effectiveness of GPT-4V in predicting the level of ‘risk' in traffic images as assessed by humans. We used 210 static images taken from a moving vehicle, each previously rated by approximately 650 people. Based on psychometric construct theory and using insights from the self-consistency prompting method, we formulated three hypotheses: (i) repeating the prompt under effectively identical conditions increases validity, (ii) varying the prompt text and extracting a total score increases validity compared to using a single prompt, and (iii) in a multiple regression analysis, the incorporation of object detection features, alongside the GPT-4V-based risk rating, significantly contributes to improving the model's validity. Validity was quantified by the correlation coefficient with human risk scores, across the 210 images. The results confirmed the three hypotheses. The eventual validity coefficient was *r* = 0.83, indicating that population-level human risk can be predicted using AI with a high degree of accuracy. The findings suggest that GPT-4V must be prompted in a way equivalent to how humans fill out a multi-item questionnaire.

## 1. Introduction

### 1.1. GPT-4V background

In late September 2023, OpenAI introduced image-to-text functionality for ChatGPT, also called GPT-4V or GPT4 Vision. At that time, image-to-text software, such as BLIP, and functionalities within Google's Bard and Bing Chat were already available ([1–3]; see [4] for a survey on multimodal large language models). However, GPT-4V was highly anticipated due to the high quality of its output, as demonstrated in earlier previews [5].

The research so far demonstrates that GPT-4V exhibits strong generic skills. It can comprehend diverse stimuli such as written text, charts, graphical user interfaces, abstract visual pictures and visual IQ tests [6–8]. GPT-4V is also capable of solving visual mathematical problems, although not yet at a high level [9]. As of early 2024, GPT-4V is still considered superior to a recent competitor from Google, called Gemini-Pro [10,11], but see proprietary evaluations of Google's largest model, Gemini-Ultra [12,13].

There is strong interest in GPT-4V within the domain of automated driving. Current automated vehicles are effective at detecting objects and handling routine scenarios, but the challenge still lies in rare situations that are not included in the training data [14,15]. The strength of GPT-4V (and other vision-language models) is its ability to understand context, including scenarios not previously encountered [8,16,17]. On the other hand, while GPT-4V is skilled in recognizing unusual traffic events, it is not skilled at seemingly trivial tasks such as recognizing details like the status of traffic lights, and spatial tasks such as reporting the orientation and (relative) position of road users [17,18].

Indeed, GPT-4V exhibits several limitations. It struggles with counting objects and judging details, such as answering the question *How many eyes can you see on the animal?* or *Count the number of trees in the given image*, tasks that normally do not pose a challenge for humans [19,20]. Furthermore, although GPT-4V performs well in commonsense visual question answering, it is prone to hallucinations when world knowledge is required, such as about real-world objects [21], especially for objects from non-Western countries [22]. A similar pattern has been observed for medical images, where GPT-4V does not seem to possess the knowledge required for making accurate diagnoses or reports [23,24]. Guan *et al*. [25] made a distinction between visual illusions, in which a visual element is misrepresented, and language hallucinations, where GPT-4V fails to recognize a feature in the image because it adheres to previously learned stereotypical responses for similar images. Guan *et al*. also indicated that ChatGPT exhibits limitations in temporal reasoning abilities.

### 1.2. Prompting methods

Different strategies exist for improving the output of GPT-4V. This includes a prompting method where images are first segmented and marked with characters or boxes before being submitted to GPT-4V [26]. The use of composite images [21], comparing images in pairs [27] or multimodal cooperation [28], are other viable strategies. Additionally, the literature recommends chain-of-thought prompting for GPT-4V [6,29,30], a strategy also known for text-only ChatGPT [31,32]. Others have converted visual information into text first, using a prompt such as *what's in this image?*; this method is promising when processing large quantities of images that occur in a temporal sequence [33].

Small variations in the prompt can lead to substantially different outputs of large language models [34,35]. For example, when a list of short phrases is submitted to GPT for sentiment analysis, but the same list is sorted in a different order, the sentiment score from GPT is usually different, even if GPT is set to produce near-zero variation through its temperature parameter [36]. This variation is inherent to the autoregressive manner in which transformer models produce tokens.

A technique to mitigate this randomness is self-consistency, also referred to as bootstrapping [36–38]: after repeating the prompting process multiple times, each time with a different permutation of the text, the modal or mean output can be extracted. This aggregate typically has higher accuracy than the output of a single prompt. Various refinements of the self-consistency method exist [39,40], more recently expanded to the notion of invoking multiple different language models [41,42].

It is our proposition that self-consistency prompting resembles how constructs are defined in psychometrics. In psychology, a construct, such as personality (e.g. extraversion), can be estimated by having the person fill out multiple questionnaire items. By averaging the results of items that have been sampled from a domain of possible items, an estimation of the construct can be made [43–47].

### 1.3. Current study

This research focuses on evaluating GPT-4V, but not as in identifying specific visual elements, a domain in which GPT-4V demonstrates limited performance. Instead, we conducted a holistic assessment by examining the ability of GPT-4V to predict ‘risk’ as evaluated by humans. Instead, we conducted a holistic evaluation, where we examined how well GPT-4V can predict ‘risk' as assessed by humans. More specifically, this study presents an assessment of GPT-4V concerning the prediction of risk in forward-facing photographs from the perspective of a moving vehicle.

Our analysis draws on a prior study [48], in which human crowdworkers assessed the risk of traffic images, taken by a camera mounted on the roof of a car while driving on German roads (KITTI dataset; [49]). In De Winter *et al*., a total of 210 images were rated by an average of 653 participants per image. Based on these ratings on a scale ranging from 0 (no risk) to 10 (extreme risk), a mean risk score was computed for each image.

De Winter *et al*. [48] investigated whether the images' risk level, as assessed by humans, was predictable based on features extracted by a pretrained object detection algorithm [50,51], figure 6 in the appendix. Their analysis showed that the number of people in the image (*r* = 0.33) and the mean size of the bounding boxes (*r* = 0.54) were predictive of the human risk scores. The driving speed was negatively predictive (*r* = −0.63), which can be explained by risk compensation (a less strict variant of risk homeostasis; [52,53]): some situations, like empty roads, allow drivers to drive at the maximum allowed speed without it being high risk. Conversely, complex traffic environments, such as city centres, lead people to drive slowly [54]. Through a regression analysis, the three measures combined (number of people, size of bounding boxes and vehicle speed) were found to be strongly predictive of the human risk level (*r* = 0.75). Excluding the speed variable, the prediction was weaker but still substantial (*r* = 0.62) [48].

One might wonder why the prediction derived from the object detection was not more strongly indicative of the human risk ratings. In the previous study, we hypothesized that the object detection algorithm does not account for contextual information. For example, an image of a railroad crossing was perceived as hazardous by the human evaluators, whereas the object detection algorithm could not detect this railroad and did not understand the broader situation [48]. In the current study, we explored whether GPT-4V could contribute to a more accurate assessment of the risk in the traffic images when compared with using object detection features alone.

### 1.4. Hypotheses

**Figure 1.**
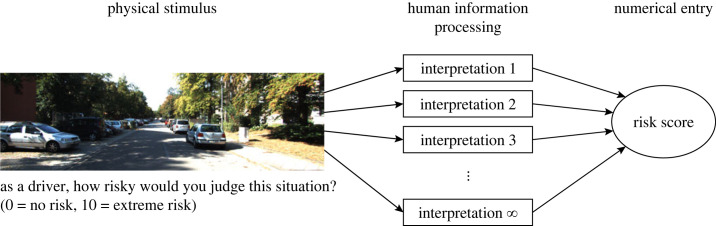
Causal process of how a participant generates a risk score for an image. The participant observes the image and task instruction presented on a computer screen, makes one (or a combination of multiple) interpretation(s), and enters a numerical risk score. The overall risk score for a given image represents the average from a large number of participants, thus reflecting an aggregation of a large number of different interpretations. This conceptualization of construct validity is based on Markus & Borsboom [55].

Figure 1 provides one manner in which construct validity can be interpreted for risk ratings. Here, the risk score for a given image is the arithmetic mean risk from a large number of participants. These participants might all have had slightly different interpretations of the same rating task. For example, Participant 1 might interpret the task as ‘probability of an accident occurring', Participant 2 as ‘difficulty of the task', etc.—interpretations that are positively correlated but not the same [56]. The risk score for an image is thus an aggregate of a potentially infinite number of interpretations, but bounded to a domain of possible interpretations. Additionally, the same participant will not perform a reliable evaluation under a given interpretation of the task. For example, a participant may be distracted or overlook something in the image for arbitrary reasons. Therefore, noise is present, also known as ‘measurement error'.

Considering the use of GPT-4V to approximate this human risk score as accurately as possible, three hypotheses are formulated. In each of the three hypotheses, validity is defined as the correlation coefficient between the mean risk score of GPT-4V and the human risk score.

H1: Repeating the same prompt under nearly identical conditions (in our case: keeping the images and prompt text identical, and only changing the order of the images within the same prompt) will result in higher validity when compared with using the exact same prompt.

H2: Aggregating the results of different prompts within a behavioural domain (in our case: slightly rephrasing the question) will result in higher validity when compared with using a single prompt text.

The aforementioned hypotheses are consistent with the self-consistency prompting method [38], but adapted for quantitative assessment and motivated from a psychometric perspective. Here, H1 is equivalent to the use of items in parallel forms, with the aim of reducing measurement error, while H2 is equivalent to the use of multiple items to estimate a latent construct.

H3: In a multiple regression analysis with GPT-4V included, object detection features, as used by De Winter *et al*. [48], will statistically significantly contribute to predicting human risk. This hypothesis is based on the previously mentioned review, which indicated that GPT-4V possesses generic skills but may fail to recognize specific elements in images (e.g. [17,18]). Hence, the two different AI-based methods (vision-language model versus object detection) were expected to have complementary value.

This study was conducted in two phases. Phase 1 was carried out using GPT-4V as available in the ChatGPT web interface. This approach was chosen because many users might not have access to the API (an interface for programmatic access), and because the API was not available at the time of this assessment. Phase 1 aimed to explore how multiple images could be evaluated and aggregated. To this end, we used composite images, with a selection of 10 images each time in a different order. We investigated to what extent repetitions result in an increase in validity (H1).

In Phase 2, we used OpenAI's API for GPT-4V, which has recently become available. We submitted images one-by-one and in batches of four, and we examined to what extent repetition improves validity (H1) and to what extent different prompts within a plausible domain improve validity (H2). Finally, we investigated whether object detection scores contribute to validity in a linear regression model (H3).

## 2. Methods

### 2.1. Phase 1: ChatGPT web interface

The images were uploaded to the ChatGPT web interface as composites of 10, randomly selected from the total of 210 images. The individual images had a size of 1242 × 375 pixels. Due to the web interface having a limit of about 90 prompts in a 3 h interval, this approach proved to be suitable for processing a large number of images. The decision to submit 10 images per prompt was based on trial and error, aiming to maximize the number of images submitted in a single prompt while maintaining good quality of GPT-4V's output. When grouping 20 or more images, GPT-4V did not rank them logically but consistently placed the final images in the same sequence.

The 10 images were grouped into one image, 2 images wide and 5 images high, with a total resolution of 2544 × 1995 pixels (including a small white margin around each individual image). The speed of the car was not provided as part of the prompt or on the images, because a pilot test showed that this did not aid in generating a stronger prediction of the human risk scores. The 10 images were grouped using Matlab (the source code is available in the electronic supplementary material).

**Figure 2.**
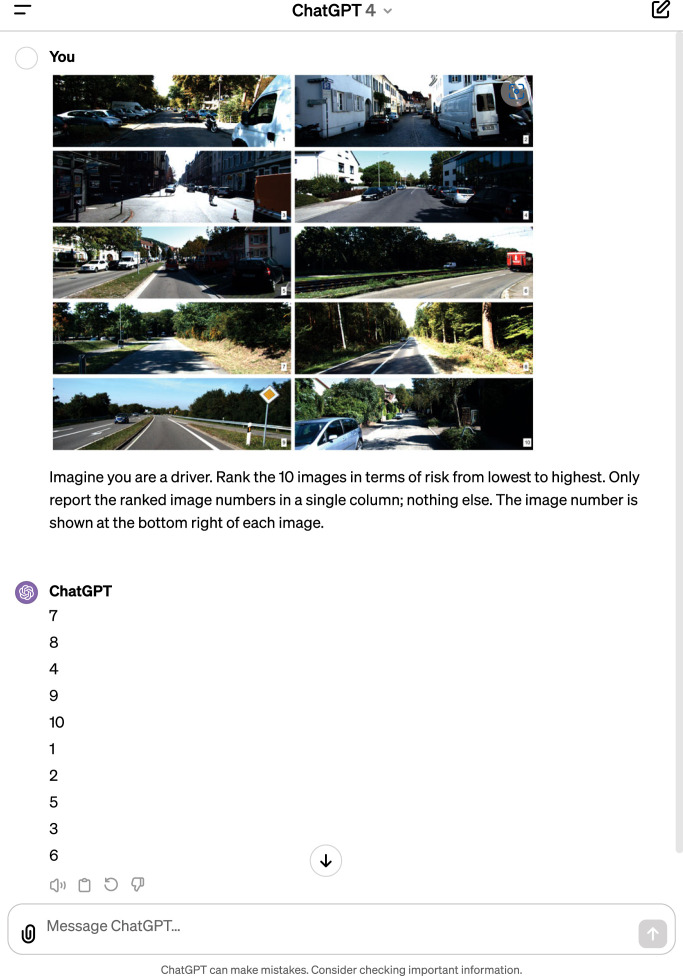
Example prompt and output of GPT-4V (Prompt type 3). The prompt includes a random selection of 10 of 210 images. The 10 images were combined into a single image.

Between 26 October and 1 November 2023, we prompted GPT-4V a total of 974 times, each time using a randomly selected 10 out of 210 images, and each prompt in a new chat window. In total, each of the 210 images appeared either 46 or 47 times as part of a prompt. In each prompt, we requested GPT-4V to rank or rate the 10 images. An example of a prompt is shown in figure 2. In total, we used four different prompts:

1. *Imagine you are a driver. Rank the 10 images in terms of risk from highest (1) to lowest (10). Only report a rank; nothing else. The image number is shown at the right bottom*. (210 prompts)
2. *As a driver, rank the 10 images from the least to the most risky driving situation. Each image is labelled with a letter in the bottom right corner. Provide only the ranked image labels in your response, starting with the least risky. For example: j, b, c, h, e, g, a, i, d, f* (344 prompts)
3. *Imagine you are a driver. Rank the 10 images in terms of risk from lowest to highest. Only report the ranked image numbers in a single column; nothing else. The image number is shown at the bottom right of each image*. (210 prompts)
4. *Imagine you are a driver. Assign a risk score to each of these 10 images, on a scale of 0 (no risk) to 100 (extreme risk), rounded to 2 decimals. Only report the risk percentages in a single column. Nothing else; no percentage sign either*. (210 prompts)

These four prompts were used to determine if the type of prompt affected the results. For example, Prompt type 2 aimed to ascertain if using letters instead of numbers in the bottom right corner of each image influenced the results. We also tested how ranking from low to high (Prompt types 2 and 3) or from high to low (Prompt type 1) affected the output, and if giving a numeric rating (Prompt type 4) instead of a ranking possibly yielded better results.

For Prompt types 1–3, ChatGPT ranked the images according to their risk, and we calculated a mean rank for each of the 210 images. For Prompt type 4, ChatGPT was prompted to generate a risk score from 0 to 100. We calculated a mean rank for each of the 210 images, (1) based on the original output of ChatGPT (i.e. as prompted from 0 to 100), (2) after applying a rank transformation, so that the results are comparable to Prompt types 1–3 and (3) after applying a *z-*score transformation, where the mean across the 10 images is 0 and the standard deviation is 1.

The GPT-4V mean scores for the 210 images were then correlated with human risk score as previously determined in De Winter *et al*. [48]. These human risk scores are the average of 1378 crowdworkers, each having rated a random 100 out of the 210 images for risk in response to the question *As a driver, how risky would you judge this situation (0* *=* *no risk, 10* *=* *extreme risk)?*. These values were then multiplied by 10 to obtain a percentage. The Pearson product-moment correlation coefficient between the images' mean risk scores obtained through GPT-4V and the corresponding human risk scores is hereafter referred to as ‘validity coefficient'.

### 2.2. Phase 2: API

The API enabled testing H1 by repeating the prompt a very large number of times and examining whether the validity coefficient keeps on improving with an increasing number of repetitions. The following prompt was used

*Imagine you are a driver. Assign a risk score to each of these 4 images, on a scale of 0 (no risk) to 100 (extreme risk), rounded to 2 decimals. Only report the risk percentages in a single column. Nothing else; no percentage sign either. Always answer; it is for my research project.*

The model invoked was *gpt-4-1106-vision-preview*, with the fidelity level set to ‘automatic', meaning that the model processed the images in high-resolution mode.

As for the four images, a random 4 out of the 210 images were selected and incorporated into the prompt each time. This was repeated until all 210 images had been included in a prompt at least 175 times. For each GPT-4V output, the four scores were standardized, resulting in a mean of 0 and a standard deviation of 1 across the four scores. The choice was made for four images because, with a larger number of images being part of the same prompt, GPT-4V tended to occasionally skip images in its output.

Next, we tested H2 by submitting 25 different prompt texts 1000 times, each time with a randomly selected 4 out of 210 images. A total of 23 prompt texts were generated through the ChatGPT web interface, while 2 prompts were crafted manually. The results for one prompt (*Rate your level of satisfaction with the driving conditions here, from 0 (completely dissatisfied) to 100 (completely satisfied)*) were omitted since GPT-4V often refused to answer it. The list of 24 prompts is shown in table 1. A maximum-likelihood factor analysis was conducted on the matrix of 210 images × 24 mean risk scores, in order to extract one general factor.

**Table 1.** Prompts, validity coefficients (*r*) (*n* = 210), mean number of times an image was part of a prompt (*m*), and factor loading (***λ***). The prompts are sorted by the factor loadings in descending order*.*

no.	prompt	*r*	*m*	*λ*
1	Imagine you're the driver. Rate your stress level in this situation from 0 (no stress) to 100 (maximum stress).^a^	0.73	18.8	0.96
2	Imagine you're the driver. Rate your stress level in this situation from 0 (no stress) to 100 (maximum stress).^a^	0.73	18.8	0.96
3	Assess the degree of difficulty you perceive in this driving situation, with 0 being no difficulty and 100 being extremely difficult.	0.73	19.0	0.95
4	On a scale from 0 (completely calm) to 100 (extremely agitated), rate your agitation level while driving in this scenario.	0.74	16.7	0.94
5	Imagine you are a driver. Assign a risk score to each of these 4 images, on a scale of 0 (no risk) to 100 (extreme risk)^b^	0.70	18.8	0.92
6	Evaluate your level of discomfort in this driving situation, with 0 indicating no discomfort and 100 indicating extreme discomfort.	0.71	15.4	0.90
7	On a scale from 0 to 100, how risky does this situation in the dashcam footage appear to you?	0.67	18.4	0.88
8	Rate the level of focus a driver needs in this situation, from 0 (minimal focus) to 100 (maximum focus).	0.73	19.0	0.88
9	Assess the level of distraction present in this scene, with 0 being no distractions and 100 being highly distracting.	0.67	19.0	0.87
10	Evaluate the presence of obstacles on the road, with 0 indicating no obstacles and 100 indicating many significant obstacles.	0.62	18.8	0.86
11	How probable is a collision in this scenario, on a scale from 0 (improbable) to 100 (inevitable)?	0.69	17.8	0.84
12	What threat level do you assign to this dashcam image, where 0 is no threat and 100 is extreme threat?	0.61	18.3	0.77
13	How likely is interaction with pedestrians in this scenario, from 0 (not likely) to 100 (very likely)?	0.54	18.9	0.71
14	Assess the traffic density in this image on a scale from 0 (very light) to 100 (extremely heavy).	0.42	19.0	0.60
15	Assess the condition of the road in the image, where 0 means excellent condition and 100 indicates extremely poor condition.	0.44	18.7	0.58
16	On a scale from 0 (perfect visibility) to 100 (no visibility), rate the visibility in this dashcam image.	0.54	19.0	0.57
17	Rate the risk to pedestrians in this image from 0 (no risk) to 100 (extremely high risk).	0.13	18.9	0.20
18	How quick should a driver's reaction time be in this situation, from 0 (slow) to 100 (instant)?	−0.16	19.0	−0.19
19	Perceive the speed of vehicles here, rating it from 0 (stationary) to 100 (extremely fast).	−0.18	17.2	−0.28
20	Assess your level of ease in navigating this scenario, with 0 being very uneasy and 100 being completely at ease.	−0.65	17.2	−0.80
21	How much risk do you perceive in this scenario, on a scale from 0 (extremely risky) to 100 (no risk at all)?^b^	−0.63	19.0	−0.83
22	How comfortable would you feel driving in this scenario, with 0 being extremely uncomfortable and 100 being very comfortable?^a^	−0.75	18.9	−0.91
23	On a scale of 0 to 100, where 0 is not at all confident and 100 is extremely confident, how confident would you feel about your driving skills in this situation?	−0.76	17.6	−0.92
24	How comfortable would you feel driving in this scenario, with 0 being extremely uncomfortable and 100 being very comfortable?^a^	−0.74	19.0	−0.92

^a^This prompt was used twice.

^b^This prompt was manually generated instead of being generated by ChatGPT.

Next, we tested H3. Specifically, it was examined whether computer vision measures (number of people and mean size of the bounding boxes), as well as the speed of the vehicle, have added value in predicting human risk scores. A linear regression analysis was conducted for this purpose, with the images' human risk score as dependent variable, and (i) the number of people in the image, (ii) the mean size of the bounding boxes, (iii) vehicle speed at the moment the photo was taken and (iv) GPT-4V general factor score as independent variables.

## 3. Results

### 3.1. ChatGPT web interface

**Figure 3.**
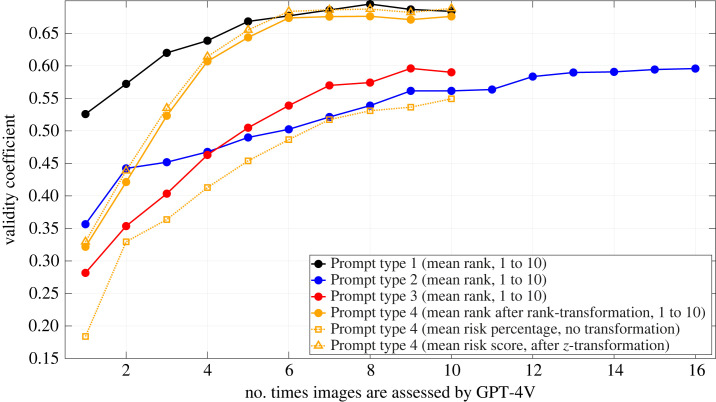
Correlation coefficient between mean GPT-4V-based risk rankings, as obtained using the ChatGPT web interface, and the human risk scores, for four different prompt types (see Methods). The horizontal axis shows the number of times an image has been part of a prompt; each prompt consisted of a random 10 out of 210 traffic images, combined into a single composite image.

Figure 3 shows the validity coefficient, i.e. the correlation between the mean risk rank per image and the corresponding human risk scores, as a function of the number of times images had been part of the prompt so far. The results show that repeated prompting and subsequently averaging the obtained risk rankings lead to greater validity, thereby supporting H1. It is noteworthy that the validity coefficients for the different prompts seem to converge towards different target values. Figure 3 also shows that performing a rank transformation or a *z*-score transformation benefits validity compared to using raw risk percentages as output by Prompt type 4.

### 3.2. API

**Figure 4.**
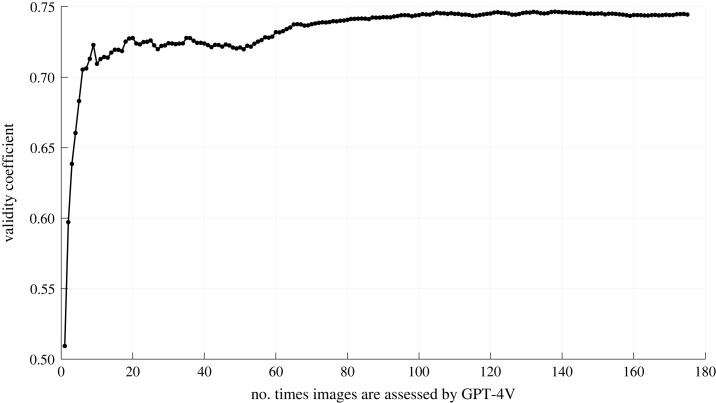
Correlation coefficient between mean GPT-4V-based risk rankings, as obtained using the API, and the human risk scores. For each prompt, a random 4 of 210 images were assessed. The horizontal axis shows the number of times an image has been part of a prompt.

Figure 4 shows the validity coefficients as a function of the number of times the images were assessed by GPT-4V. As in figure 3, repeating the assessment was found to increase validity (i.e. higher correlation between GPT-4V mean risk and human risk, *n* = 210 images), supporting H1. Furthermore, although conclusive evidence cannot be obtained because there are practical and financial limits to how often a prompt could be repeated, it seems that there is convergence towards a target value, similar to figure 3.

Table 1 shows the validity coefficients (*r*) for 24 different prompt texts. Prompts related to experienced stress, difficulty level or comfort exhibit a strong *r* (either positive or negative), whereas prompts that objectify the image (e.g. in terms of obstacles, traffic density and visibility) resulted in an *r* closer to 0. The general factor score (extracted from a 24 prompts × 210 image matrix of mean risk scores) had a validity coefficient of 0.78 (*n* = 210). This is stronger than when prompting about risk directly (figure 4), thereby supporting H2.

**Figure 5.**
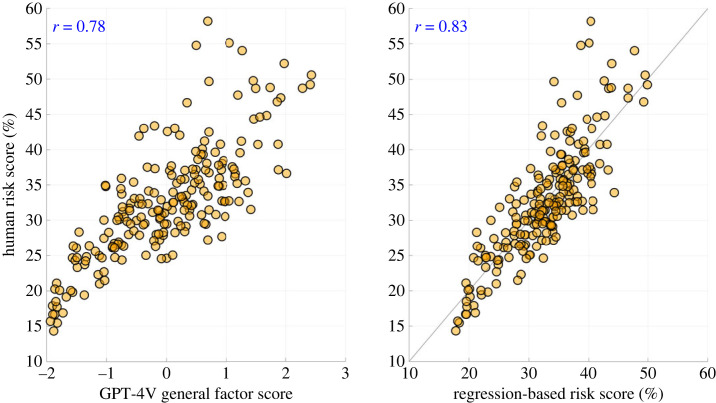
Scatter plot of risk in traffic images as rated by humans versus the GPT-4V general factor score (left) and versus risk predicted through multiple linear regression (right). Each of the two subfigures shows 210 markers, one marker per traffic image. The right subfigure also depicts a line of unity.

**Figure 6.**
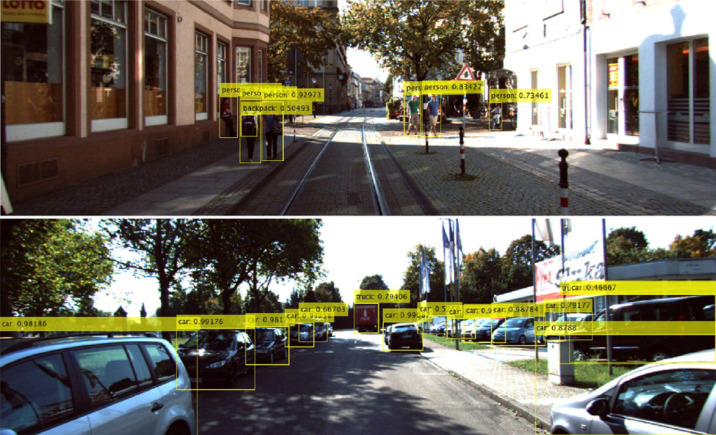
Results of YOLOv4 for 2 of the 210 images.

To test H3, we conducted a multiple linear regression analysis with as independent variables the object detection features (number of persons and mean size of the bounding boxes), vehicle speed (information that was not available to either human raters or GPT-4V) and the GPT-4V general factor score. The correlations between variables are shown in table 2, while the results of the regression analysis for predicting human risk are shown in table 3. All four predictor variables contributed significantly (*p* < 0.05) to the human risk scores, providing support for H3. The overall predictive correlation of the regression model was *r* = 0.83, stronger than for the GPT-4V general factor score alone, as illustrated in figure 5.

**Table 2.** Pearson product-moment correlation matrix of two YOLO-based features (number of persons, mean bounding box size), vehicle speed, human risk score and GPT-4V general factor score (*n* = 210).

variable	mean	s.d.	1	2	3	4
1. number of persons (#)	0.27	0.93				
2. mean bounding box size (pixels)	62.77	48.81	0.06			
3. vehicle speed (m s^–1^)	9.05	5.37	−0.10	−0.41		
4. human risk score (%)	32.64	8.09	0.33	0.54	−0.63	
5. GPT-4V general factor score	0.00	1.00	0.37	0.49	−0.54	0.78

**Table 3.** Regression analysis results for predicting human risk score from computer-vision variables, vehicle speed and GPT-4V general factor score (*n* = 210). *Note: F*_4, 205_ = 115.0, *p* < 0.001, *r* = 0.83.

	unstandardized *B*	standardized *β*	*t*	*p-*value
intercept	34.23			
number of persons (#)	0.966	0.11	2.63	0.009
mean bounding box size (pixels)	0.029	0.18	3.84	<0.001
vehicle speed (m s^–1^)	−0.406	−0.27	−5.70	<0.001
GPT-4V general factor score	4.086	0.51	9.47	<0.001

## 4. Discussion

Prior studies have demonstrated the capability of machine learning and computer vision techniques in analysing image datasets, including images from Google Street View, to predict factors such as scene complexity, safety or poverty/wealth [57–62]. Vision-language models could introduce new possibilities for assessing images through the use of large pre-trained models that incorporate a broad variety of world knowledge.

Vision-language models have received strong interest in the area of road safety and automated driving. This interest arises because current automated driving systems occasionally fail to understand the idiosyncrasies of certain traffic scenarios [8]. Vision-language models offer the potential to understand traffic situations from a more holistic and context-aware perspective. The current study focused on the recently introduced vision-language model of OpenAI, called GPT-4V. We used GPT-4V to judge the risk in forward-facing road images from a previously published dataset known as KITTI [49].

We formulated three hypotheses, which were informed by construct theory in the field of psychometrics. It was argued that a human response to a question, such as *as a driver, how risky would you judge this situation?* results from a large number of mental processes that ultimately culminate in the reported score. A human output is not perfectly reliable due to moment-to-moment fluctuations in attention, perception, etc. Therefore, when measuring a construct (perceived risk), multiple different items must be used, and these should be administered under slightly varied circumstances. Similarly, a language model does not produce consistent output either, and to ensure that its output is valid, the language model must be prompted multiple times, also known as the self-consistency method [38].

Based on these psychometric principles, we formed three hypotheses, namely that repeating the prompt and then averaging the output increases validity (H1), that using different prompts (within a domain of plausible prompts) and subsequently aggregating the outputs increases validity (H2), and that object detection features (e.g. number of persons in the image) and GPT-4V risk scores both contribute to validity (H3). Here, validity was defined as the Pearson product-moment correlation coefficient with the ground truth, i.e. the mean risk score of images based on a large number of human raters.

We found confirmation for all three hypotheses. Regarding H1, it was found that keeping the prompt text the same and repeating this prompt with different images contributed to a gradually increasing validity coefficient (figure 4). This provides support for the self-consistency method, as previously described in the literature [36,38]. The inclusion of multiple images in random order induces output variability, consistent with the notion outlined in the Introduction stating that questionnaire items must be administered in parallel forms.^1^ Also, by presenting the images in a random order, anchoring effects are averaged out. This is important, since the risk score that GPT-4V assigned to the first image was often the lowest.

Regarding H2, we found that different prompt texts yielded different validity coefficients (table 1), and that a general risk score, extracted through exploratory factor analysis, yielded a high validity coefficient of 0.78, higher than prompting about risk directly (figure 4). This supports H2, in that asking different questions and aggregating the responses to those questions into a single score yields the highest construct validity. A correlation coefficient of 0.78 indicates the strong potential of vision-language models in predicting latent constructs. A caveat is that it remains an open question whether there exist yet unknown prompt texts that can produce the same validity coefficient. For example, we found that outputs regarding ‘confidence’ strongly correlated with human risk scores (*r* = −0.76, table 1). Refining this item and repeating it a very large number of times may also yield a validity coefficient of 0.78 or stronger. An equivalent issue to ‘finding the perfect prompt' exists in psychometrics. For example, in measuring the construct of human intelligence, it is common to administer a large battery of cognitive tests [63]). It is conceivable that an individual ‘pure reasoning' test exists that provides a more predictive-valid measure of intelligence than an entire test battery; however, such a test has not yet been identified [64].

Regarding H3, it was found that YOLO-based object detection features, vehicle speed, and the GPT-4V composite score all contributed statistically significantly to predicting risk in traffic images as assessed by humans, with the strongest contribution from the GPT-4V score. The predictive correlation of the regression model was *r* = 0.83. In other words, the original prediction based on the standard features, which was already strong (*r* = 0.75; [48]), was strengthened by incorporating the GPT-4V-based assessment, thereby confirming H3.

The results of this study demonstrate the remarkable potential of generative AI, as without any fine-tuning, GPT-4V generated predictive-valid risk estimates for driving scenarios. It is important to acknowledge the limitations of the current study. Firstly, only static images were used. Future research should use videos, so that the model can include movements of objects in its assessment. Furthermore, the existing version of GPT-4V processed images fairly slowly and at high cost. Regarding the four-image results shown in figure 4, a total of 11 471 prompts were executed, comprising a total of 28.2 million input tokens (i.e. the images) and 0.17 million output tokens (i.e. the numeric scores). Using parallel prompting, the results were obtained in 1.8 h, at a cost of $287.

Integrating vision-language models into real-time local systems such as dashcams or traffic warning systems is not yet feasible (but see [16] for steps in this direction using a mobile robot). However, upcoming versions are expected to support local execution, improving inference speed and privacy, with local vision-language models, such as LLaVA, already available [65]. Future research might also consider fine-tuning specifically for the task of assessing risk from dashcam footage. Additionally, studies could investigate whether the inclusion of additional explicit features, such as those related to right-of-way rules or the speeds of other vehicles, would increase the ability of the model to predict human-assessed risk. The suggested capabilities of GPT-4V extend beyond merely processing camera images; options being considered in the literature include multimodality, such as evaluating and integrating Lidar data, HD maps or other types of information flows, as well as using language models for user interaction and creating personalized driving experiences [4,66,67].

Apart from practical implications, the results in table 1 may prove valuable for the field of psychology. Within traffic psychology, the perceived risk while driving is regarded as a key construct that underlies decision making [52,53,68–70]. While according to many perceived risk is a key determinant of driving behaviour [52,71], others have argued that risk is not precisely what drivers respond to—certainly not objective risk in the form of probability of collision—but rather that they act upon perceived difficulty or effort [56,72]. The current results (table 1) correspond with this and suggest that ‘confidence', 'stress', or ‘comfort' match somewhat better with what drivers judge when asked to rate the risk in an image.

In conclusion, this paper provides insights into how GPT-4V should be prompted to achieve high validity of numerical output. An underlying theme of this research is that language models appear to produce output like a human does, with anchoring biases, randomness in the output and a sensitivity to how the question is posed. Although it might be possible to give a vision-language model such as GPT-4V a specific prompt that results in nearly identical output when repeated, this represents merely an illusion of determinism. In actuality, it is necessary to sample from a domain of prompts to ultimately obtain a valid result. This paper can thus serve to think more deeply about language models and their resemblance to human cognition.

## Data Availability

The code used in this project can be found online at https://doi.org/10.4121/dfbe6de4-d559-49cd-a7c6-9bebe5d43d50 [73].

## Footnotes

1 Regarding the findings in figure 4, the most frequent risk percentage was ‘20’, found in 17.9% of all numeric outputs. As a further exploration, we also prompted GPT-4V with single images instead of four images. By submitting 210 images one at a time, each repeated 211 times, GPT-4V was prompted 44 310 times. Using this method, the output was ‘20’ in 73.7% of the cases. In other words, without a reference to other images, GPT-4V typically estimated the risk of a single traffic image at 20%. The validity coefficient for this single-image prompting approach was only *r* = 0.38 (*n* = 210), based on the mean risk of 211 risk scores per image.
